# The Amyloid Region of Hfq Riboregulator Promotes DsrA:*rpoS* RNAs Annealing

**DOI:** 10.3390/biology10090900

**Published:** 2021-09-12

**Authors:** Florian Turbant, Pengzhi Wu, Frank Wien, Véronique Arluison

**Affiliations:** 1 Laboratoire Léon Brillouin LLB, CEA, CNRS UMR12, Université Paris Saclay, CEA Saclay, 91191 Gif-sur-Yvette, France; flo.turbant@gmail.com; 2 Department of Biology, ETH Zürich, 8093 Zürich, Switzerland; pengzhi.wu@bc.biol.ethz.ch; 3 Synchrotron SOLEIL, L’Orme des Merisiers, Saint Aubin BP48, 91192 Gif-sur-Yvette, France; 4 UFR Sciences du Vivant, Université de Paris, 75006 Paris, France

**Keywords:** bacterial amyloid, functional amyloid, RNA chaperone, RNA/RNA annealing, stress adaptation, DsrA noncoding RNA

## Abstract

**Simple Summary:**

RNA:amyloid protein interactions have been observed in the past few years. Nevertheless, the molecular basis and physiological implications of these interactions are still poorly understood. Here we focus on a bacterial amyloid protein, Hfq. This protein is a pleiotropic bacterial regulator that mediates many aspects of RNA metabolism. The protein notably mediates mRNA stability and translation efficiency by using stress-related small noncoding regulatory RNAs. This regulation contributes to bacterial adaptation to stresses. Our results show that the amyloid region of Hfq significantly influences the efficiency of annealing between DsrA small noncoding RNA to its target mRNA. This unexpected result opens perspectives for a novel physiological role of amyloids, including those associated with neurodegenerative diseases.

**Abstract:**

Hfq is a bacterial RNA chaperone which promotes the pairing of small noncoding RNAs to target mRNAs, allowing post-transcriptional regulation. This RNA annealing activity has been attributed for years to the N-terminal region of the protein that forms a toroidal structure with a typical Sm-fold. Nevertheless, many Hfqs, including that of *Escherichia coli*, have a C-terminal region with unclear functions. Here we use a biophysical approach, Synchrotron Radiation Circular Dichroism (SRCD), to probe the interaction of the *E. coli* Hfq C-terminal amyloid region with RNA and its effect on RNA annealing. This C-terminal region of Hfq, which has been described to be dispensable for sRNA:mRNA annealing, has an unexpected and significant effect on this activity. The functional consequences of this novel property of the amyloid region of Hfq in relation to physiological stress are discussed.

## 1. Introduction

Bacteria are adapted to function in their normal physiological environment. Any change in environmental conditions such as temperature, pH, nutrients starvation, salts, and oxidation inflict stresses on bacteria [1,2]. Many regulatory systems help to respond to these changes and post-transcriptional regulation of mRNA translation and stability provides a rapid and efficient mechanism [3,4,5]. The Hfq protein, a chaperone for small noncoding RNAs (sRNAs), is considered a core component of a global post-transcriptional network in bacteria [6,7,8,9]. Hfq was discovered as a host factor required for replication of the bacteriophage Qβ in *Escherichia coli* [10,11]. Approximately 50% of sequenced bacterial genomes contain at least one gene coding for Hfq [12]. As a global regulator of *E. coli* metabolism, deletion of the *hfq* gene can cause pleiotropic effects, such as decreased growth rate, maladaptation to stress, altered cellular morphology, and increased cell length [13,14].

Hfq is a member of the Sm and Sm-like family of RNA-binding proteins [15,16,17,18]. In *E. coli*, it is composed of an Sm N-terminal region (NTR, residues 1–65) which forms a homohexameric ring-shaped structure, and an intrinsically disordered C-terminal region (CTR, residues 66–102) [18,19,20]. The surface of the Sm domain comprising the N-terminal α-helices is designated as the proximal face, the opposite face as the distal face, and the outer ring as the rim surface. All these surfaces of the NTR bind RNAs with different specificities and affinities [7,21,22,23]. The proximal face binds polyU sequences, which usually can be found in the Rho-independent terminators of sRNAs [19]. The distal face binds AAN triplet repeats, which are often found in the 5′-UTR of target mRNAs [24]. Recent work has shown that the rim surface is a secondary binding site for UA-rich sequences in sRNAs and mRNAs [25,26]. Therefore, the NTR (Sm) domain stabilizes the sRNAs against turnover [27,28,29] and promotes their interactions with mRNAs leading to altered stability and/or translation of these mRNA targets [30,31].

Although the functional importance of the Sm domain is well established, the function of the presumably disordered CTR is poorly understood [6]. Indeed, all Hfq 3D structures lack the CTR and include only the NTR of the protein [18,25,32,33,34,35,36]. CTR has been shown to be at the periphery of the tore [20] and prefers to locate at the proximal side [37]. It was initially described to be dispensable for sRNA-based regulation [38]. Nevertheless, more recent results indicate that it could play a role in sRNA post-transcriptional control [39]. The recent structure of the Hfq-RydC complex showed that the CTR makes distributive contacts over the surface of RydC sRNA, which suggested that the CTR may also help Hfq to recruit sRNAs [25]. The CTR was also proposed to be dispensable to accelerate RNA-pairing, but required for the release of double stranded RNA [40]. Combined integrative experimental techniques and multi-scale computational simulations also proposed that non-specific interactions between the CTR and RNA may play a dual role in a steric effect (especially at the proximal side) and recruitment (at the both sides) of RNAs [37]. The precise 3D structure of the CTR is also unclear [6,41]. Initially described as an Intrinsically Disordered Proteins (IDP) [37,42] it was recently shown to have the intrinsic property to self-assemble into long amyloid-like fibrillar structures in vitro and in vivo [43,44]. Amyloidogenesis of CTR is accelerated by DNA [45], but the effect of RNA on this amyloidogenic process is still unclear. Hfq-CTR thus belongs to the family of functional bacterial amyloids. Amyloid proteins are characterized by a β-sheet secondary structure (referred as cross-β structure [46]) and have a fibrillar morphology of ~10 nm in diameter [47]. While amyloids are associated with diseases such as Alzheimer’s [48], they have also useful features [49,50]. This is, for instance, the case of bacterial amyloids that play important and positive roles for the cell [51,52]. Bacterial functional amyloids usually contain a particularly high prevalence of alanine, asparagine, and threonine, and this applies to Hfq-CTR [53].

Among Hfq-dependent sRNAs, DsrA is one of the first sRNA regulators found to regulate translation of multiple mRNAs, such as *rpoS* [54], *hns* [55,56], *mreB* [57], and *rbsD* [58]. DsrA forms a structure with three stem-loops (SL1, SL2, and SL3) and a long linker between SL1 and SL2 (Linker 1). SL3 is a Rho-independent transcription terminator, which consists of many G-C base-pairs followed by multiple uridine nucleotides. SL2 contains a dynamic conformational equilibrium, so it can participate in base-pairing with *hns*, *mreB*, or *rbsD* mRNAs with different conformational states [59]. The SL1 and Linker 1 promote efficient translation of *rpoS* mRNA, which encodes for the stress sigma factor σ^S^, by acting as an anti-antisense RNA [54,60]. The 5′ UTR of the *rpoS* mRNA forms a large stem-loop structure that shields the ribosome binding site (*rbs,* or Shine-Dalgarno sequence) and therefore inhibits ribosome binding [54,61]. When DsrA pairs to the upstream region in the 5′-UTR of *rpoS*, it causes this stem-loop to open, releases the *rbs* and activates the translation of *rpoS* mRNA. Opening of DsrA SL1 is required for efficient DsrA:*rpoS* annealing [62]. However, the mechanism of SL1 unfolding remains unclear. During DsrA:*rpoS* annealing, Hfq recruits DsrA for base-pairing with *rpoS* mRNA [63]. The preferential Hfq-binding site of DsrA is Linker 1, while SL1 is partially destabilized by Hfq [62]. Meanwhile, recent NMR studies showed that SL1 has a very stable stem-loop structure, and cannot be significantly unfolded to base pair with the *rpoS* mRNA without Hfq at room temperature [59]. These results indicate that Hfq may play an important role in the unfolding of DsrA SL1.

Here, we used a biophysical approach, Synchrotron Radiation Circular Dichroism (SRCD), to study the effect of the Hfq-CTR on DsrA SL1 annealing to *rpoS* mRNA. Following RNA annealing using ElectroMobility Shift Assay (EMSA) is usually challenging for polymerizing proteins, as in the case of amyloids [64]. SRCD thus provides a new useful tool to observe RNA annealing in real time by amyloids (and in general by proteins that polymerize), but also to characterize annealing precisely. SRCD allows differentiation base pairing and base-stacking in a double-stranded helical transition [65]. Unexpected effects of the Hfq CTR amyloid-like region on the RNA annealing process are presented herein. This result reveals a perspective for a novel physiological role of amyloids, including those associated with neurodegenerative diseases.

## 2. Materials and Methods

### 2.1. Chemicals

All chemicals were purchased from Sigma-Aldrich (Saint-Louis, MO, USA) or Thermofisher scientific (Waltham, MA, USA).

### 2.2. Hfq CTR Peptide and Protein

Full-length Hfq and NTR were purified as described previously [66]. Hfq-CTR peptide was chemically synthetized (Proteogenix, Schiltigheim, France). This peptide corresponds to the amyloid CTR domain of Hfq (residues 64 to 102) and is referred to as Hfq-CTR throughout the manuscript. This peptide cannot be purified from *E. coli* bacteria as it is unstable when translated independently of the NTR region. The sequence of Hfq-CTR is SRPVSHHSNNAGGGTSSNYHHGSSAQNTSAQQDSEETE [43]. Before use, Hfq-CTR peptide was reconstituted in water at 20 mg/mL. We determined that the pH used in our condition (~5) was the most appropriate to form the complex with RNA. Indeed, the positive charge of the peptide at pH 5 allows its interaction with RNA, while increasing pH reduces or abolishes this interaction. We also chose to avoid the addition of salts (except those already present in RNA, protein, and peptide solutions) in order to allow a better investigation in deep-UV [45]. When the complex is analyzed in the presence of salts (or far-UV absorbing buffers), the spectral bandwidth accessible is limited, reducing the spectral information obtained [67]. We checked that presence of salts (NaCl 50 mM) does not change significantly the kinetics of annealing.

### 2.3. DsrA and rpoS sequences

**Figure 1:**
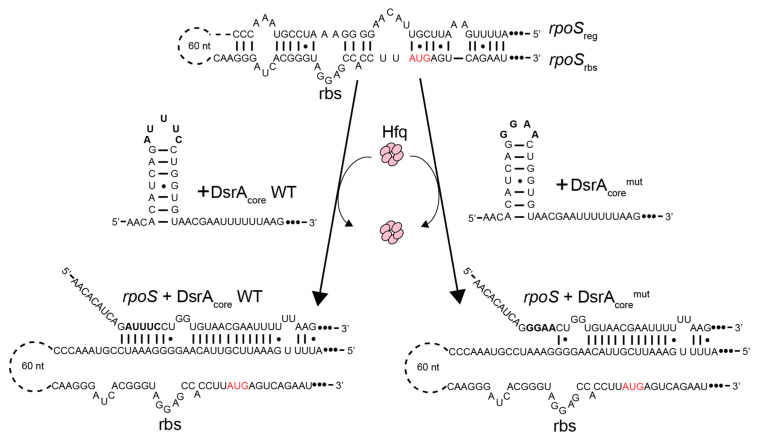
DsrA and *rpoS* fragments used in SRCD experiments. Base pairing of DsrA with the leader sequence of *rpoS* enhances its translation by exposing the ribosome binding site (*rbs*) and start codon (AUG), which are sequestered in the inhibitory stem structure. This process requires the RNA chaperone Hfq. Until now, Hfq-CTR was described as dispensable for this activity.

For SRCD analysis, we focused on the core of the RNA–RNA interactions and used DsrA and *rpoS* fragments described in Hwang et al. [68]. Two fragments of DsrA (DsrA_core_ WT and mutant) and two fragments of *rpoS* (*rpo*S_rbs_ and *rpo*S_reg_) were used (Figure 1).

Sequences of DsrA fragments were: DsrA_core_ WT (pentaloop, underlined) 5′-AACACAUCAGAUUUCCUGGUGUAACGAAUUUUUUAAG-3′, DsrA_core_^mut^ (tetraloop) 5′-AACACAUCAGGGAACUGGUGUAACGAAUUUUUUAAG-3′. Sequences of *rpoS* fragments were *rpoS*_reg_: 5′-AUUUUGAAAUUCGUUACAAGGGGAAAUCCGUAAACCC-3′; *rpoS*_rbs_: 5′-CAAGGGAUCACGGGUAGGAGCCACCUUAUGAGUCAGAAU-3′.

Before use, the oligonucleotides (Eurogentec) were heated at 90 °C for 3 min and then slowly cooled down at 20 °C to allow proper folding. Duplexes of oligonucleotides (i.e., DsrA_core_:*rpoS*_reg_, *rpoS*_rbs_:*rpoS*_reg_…) were formed using the same protocol after stoichiometric addition of the two oligonucleotides in the same tube.

### 2.4. Synchrotron Radiation Circular Dichroism (SRCD)

SRCD measurements were carried out on the DISCO beamline at SOLEIL Synchrotron as described previously [45] (proposal 20201013). Samples (~4 µL) were loaded into a CaF_2_ circular cell of 10 µm pathlength. Spectral acquisitions of 1 nm steps at 1.2 s integration time were recorded in triplicates between 320 and 180 nm. (+)-camphor-10-sulfonic acid (CSA) was used to calibrate amplitudes and wavelength positions of the experiment. Data analyses (averaging, baseline subtraction, smoothing, scaling, and spectral summations) were carried out with CDtoolX [69]. Spectra are presented in units of mdeg versus nm maintaining the same molar ratios for all presented samples. Due to the origin of absorption, spectra of mixed samples (polynucleotides + peptide) could not be standardized to ∆ε.

Two types of experiments have been performed: the effect of Hfq or CTR were tested either on individual RNA, or on duplexes such as DsrA_core_:*rpoS*_reg_, *rpoS*_rbs_:*rpoS*_reg_. For all SRCD experiments, RNA concentrations were fixed at 20 mM (while Hfq or CTR (monomeric) concentration were 0.2 or 0.4 mM, respectively (full-length Hfq concentration was lower due to its lower solubility). RNAs were in excess relative to proteins. The effect of Hfq or CTR on RNAs was analyzed at 15 °C. For kinetic measurements, an apparent catalytic kinetic constant k_cat_^app^ was determined during initial rate conditions. This constant was expressed in mdeg·M^−1^·min^−1^ and depends on protein concentration (CD units cannot be normalized to protein concentration as they are not expressed in the same unit, mdeg vs. M).

For melting curves, triplet SRCD spectra were acquired every 3 °C between 15 °C and 81 °C. Averaged SRCD values of the maximum of the peak around 180 nm are presented as a function of temperature to measure the melting point (T_m_). Note that a shift of the peak may occur in some cases and that the maximum is not always precisely at 180 nm [70] (Supplementary Materials Figure S1). A Boltzmann sigmoid equation, which assumes a two-state model, was used for fitting of melting curves: y = Bottom + (top − bottom)/(1 + e((T_m_ − x)/slope)).

### 2.5. ElectroMobility Shift Assay (EMSA)

The Electromobility Shift Assays were performed with labelled RNAs (Cy3-DsrA_core_ and Cy5-*rpoS*_reg_) or unlabeled RNA (DsrA_core_, DsrA_core_^mut^). After incubation with CTR for one hour, samples were loaded on a polyacrylamide gel (10% or 4–20%) and subjected to electrophoresis in Tris-Acetate-EDTA (TAE) buffer. The gels were scanned using a fluorescence imager.

## 3. Results

### 3.1. The Amyloid CTR Region of Hfq Triggers RNA Annealing

**Figure 2:**
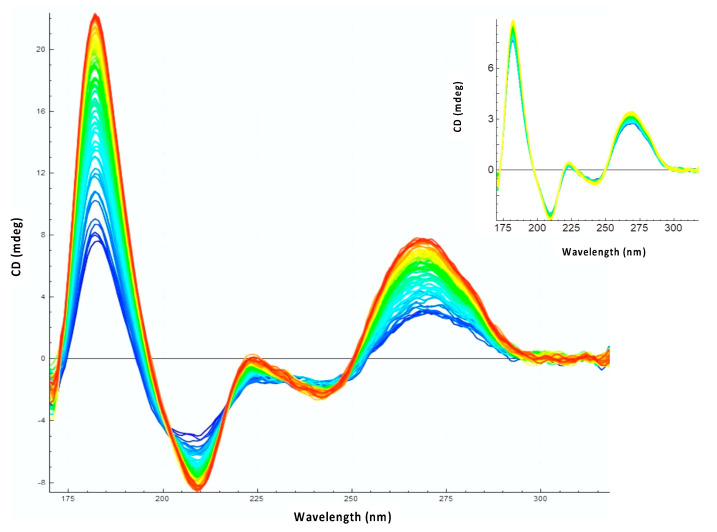
Observation of DsrA_core_ and *rpoS*_reg_ annealing by Hfq-CTR using SRCD (from blue t = 0 min to red t = 300 min). Inset: control without the protein; no significant RNA annealing occurs without the protein.

The effect of Hfq-CTR on RNA annealing was analyzed at 15 °C. This low temperature is physiologically relevant because DsrA is expressed at low temperature allowing a cold-shock response [71]. In addition, measurements at 15 °C avoid spontaneous annealing between RNA molecules occurring at 37 °C with RNA fragments (even if this spontaneous annealing at 37 °C is slow). Kinetics of annealing at 15 °C were thus longer than at 37 °C and were recorded for few hours to ensure accurate measurements. As seen in Figure 2, we clearly observe an effect of Hfq-CTR on sRNA:mRNA annealing, shown by the different increase of amplitudes observed at 180 nm and 260 nm with and without the Hfq-CTR. Indeed, the spectral region between 320 and 170 nm contains several electronic transitions of interest. Around 260 nm, the positive CD signal shows base-pairing and base-stacking [72,73]. On the other hand, the positive band at 185 nm and the negative one between 200 and 210 nm are indicative for the formation of double-stranded right-handed RNA molecules (A- and B-forms). The increase of amplitudes of these peaks signifies the formation of base pairs and right-handed double stranded RNA. In addition, denaturation of the RNA induces a shift of the spectral maximum from ~180 to ~192 nm [70]. Spectroscopically the batho-chromic shifting (red-shift) of a maximum corresponds to a change (reduction) of absorption energy of the chromophore (RNA). This shift therefore indicates a clear structural change or the existence of two states of the chromophore and can also be used to confirm the structural change of RNAs (Supplementary Materials Figure S1). Therefore, the dynamics of RNA annealing can be qualitatively and quantitatively followed. This effect of Hfq-CTR on RNA annealing was also confirmed using EMSA (Supplementary Materials Figure S2). Nevertheless, due to the high molecular weight of the complex with the amyloid form of the CTR, that stays on the top of the gel, it is difficult to discriminate solely with an EMSA the complex (i.e., triplex) CTR:Cy3-DsrA_core_:Cy5-*rpoS*_reg_ from CTR:Cy3-DsrA_core_ and CTR:Cy5-*rpoS*_reg_ duplexes. They all migrate at the same position. In addition, precise kinetics measurements are not possible using EMSA as they are with SRCD analysis.

**Figure 3:**
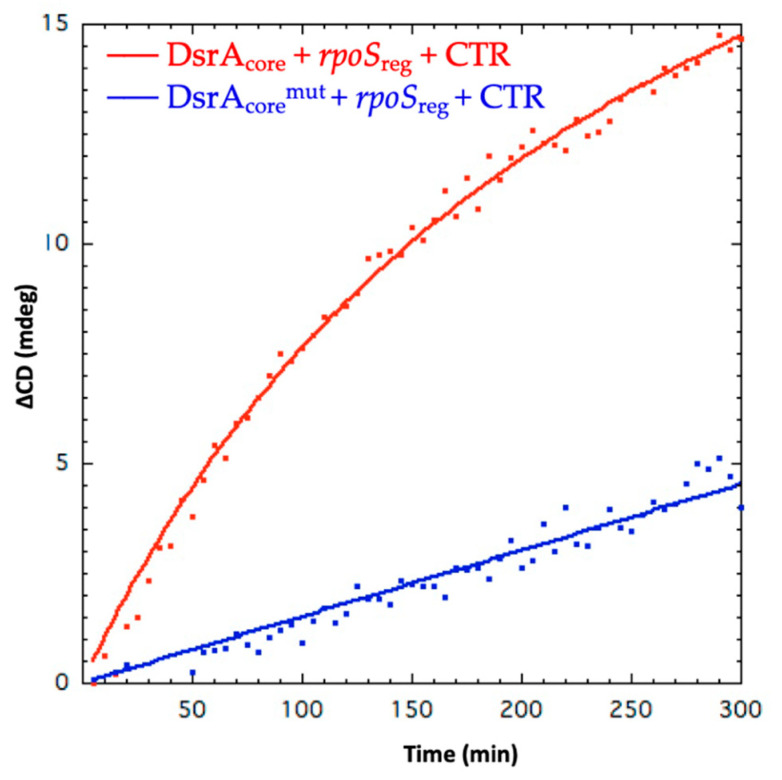
Kinetics of DsrA_core_ and *rpoS*_reg_ annealing observed at 180 nm. Red: DsrA_core_ + *rpoS*_reg_ + CTR; blue: DsrA_core_^mut^ + *rpoS*_reg_ + CTR. ∆CD refers to CD at a specific time subtracted from CD at t = 0. Kinetics constant have been measured during initial rate conditions.

SRCD kinetics of *rpoS*_reg_ annealing have been measured using SRCD for WT DsrA_core_ and for a mutated form called DsrA_core_^mut^, harboring a mutated tetraloop (GGAA vs. AUUUC pentaloop) (Figure 1). DsrA_core_ forms a stem–loop structure with a typical A-form helix and with a dynamic AUUUC pentaloop [59]. Many other pentaloops can form stable structures with unusual interactions, while AUUUC pentatloop is unstructured. To test whether the unstructured dynamic AUUUC pentaloop is important for the annealing, we replaced it with a very stable GGAA tetraloop. At 180 nm, an apparent Hfq-CTR catalytic kinetic constant k_cat_^app^ of 0.083 mdeg·M^−1^·min^−1^ for DsrA_core_ was determined compared with 0.0173 mdeg·M^−1^·min^−1^ for DsrA_core_^mut^ (Figure 3). In comparison, for full-length Hfq the kinetics constant k_cat_^app^ measured was for 0.039 mdeg·M^−1^·min^−1^ for DsrA_core_ (Supplementary Materials Figure S3); nevertheless, Hfq was two-times less concentrated than Hfq-CTR, thus CTR seems to have an activity similar to that of the full-length protein to promote DsrA_core_:*rpoS*_reg_ RNA annealing. Note that when measured at 260 nm, the k_cat_^app^ measured was 0.0257 mdeg·M^−1^·min^−1^ for DsrA_core_ and 0.0069 mdeg·M^−1^·min^−1^ for DsrA_core_^mut^, thus with a similar decrease by a factor ~5 compared with WT DsrA_core_. This suggests that the formation of base pairs/base stacking and helical structure during annealing occurs at the same rate regardless of the sequence of the loop. We also tried to analyze Hfq-NTR in SRCD experiments but failed to have this form of the protein at a concentration sufficient to measure annealing kinetics with SRCD. This shows a limit of the technique: for SRCD, short pathlengths (two to several tenths of microns) are used permitting very small loading volumes (3–4 µL). Therefore, high concentrations are chosen following Beer–Lambert law. This has the advantage of reducing overall precious sample consumption as well as extending the spectral-band down to 170 nm. With Hfq-NTR we could not reach a concentration of ~0.2 mM and increasing its concentration clearly promoted precipitation of the protein which is incompatible with SRCD measurements because it results in diffusion of the UV light. We tried to measure the annealing activity of Hfq-NTR using lower concentrations but, unfortunately, were not able to detect a significant signal change for SRCD, because the signal to noise ratios were dropping considerably below 10 in contrast to above 200 for high concentration and short pathlengths. We thus presume NTR activity is significantly lower than that of the full-length protein, even if this region is able to promote annealing in vivo [38]. This result is in agreement with the significant activity of the CTR.

**Figure 4:**
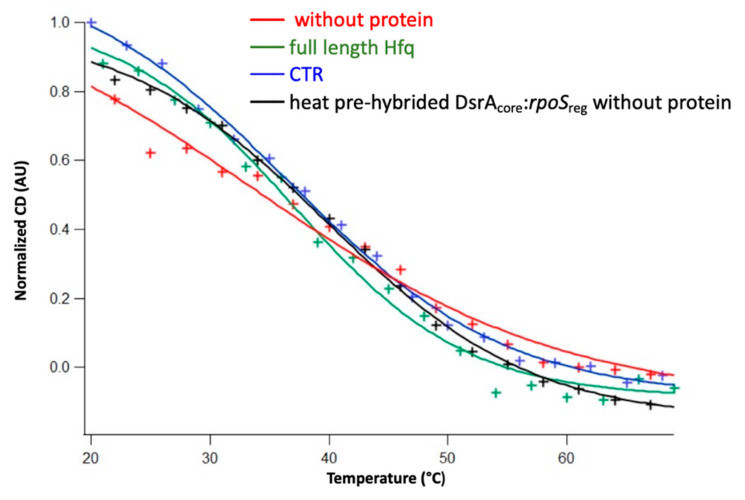
Melting curves at ~180 nm of DsrA_core_ and *rpoS*_reg_ complexes annealed without protein (red), with full-length Hfq (green), with Hfq-CTR (blue), and heat pre-hybrided DsrA_core_:*rpoS*_reg_ without protein (black), respectively. For a better comparison of various sigmoids, they were normalized between 0% and 100%. The corresponding T_m_ were 36.6 ± 0.8 °C, 37.0 ± 0.9 °C, 33.6 ± 2.2 °C, and 39.9 ± 0.4 °C.

The effect of Hfq and Hfq-CTR on the stability of the complex formed was evaluated using meting curves. As seen in Figure 4 and Table 1, the DsrA_core_:*rpoS*_reg_ complex is more stable in the presence of both Hfq and CTR. This confirms that the full protein stabilizes the sRNA:mRNA complex, but this result was unexpected for the CTR.

Note that DsrA_core_^mut^ is significantly more stable than DsrA_core_ in the absence or in the presence of CTR (Table 1). This effect is probably due to the presence of the tetraloop vs. the pentaloop. As shown in Figure 3, the effect of the replacement of the pentaloop by a tetraloop affects the rate of annealing that is decreased by a factor 5. We next analyzed the complex between CTR and DsrA_core_ or DsrA_core_^mut^ in the absence of *rpoS*.

### 3.2. Hfq-CTR Stabilizes DsrA Secondary Structure

**Figure 5:**
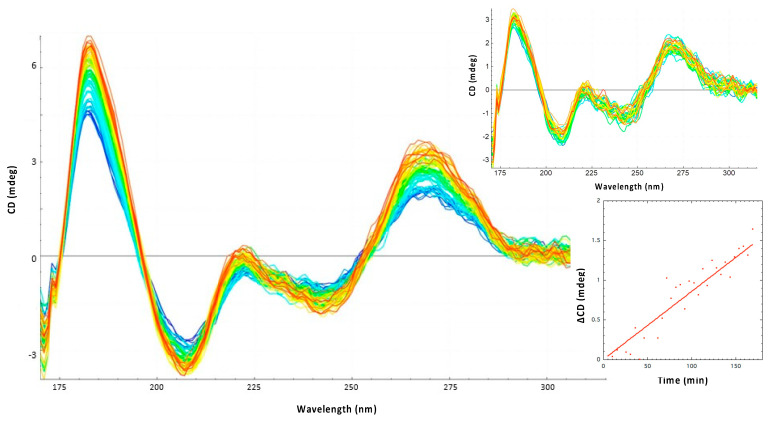
Observation of DsrA_core_ stabilization by Hfq-CTR using SRCD (from blue t = 0 min to red t = 300 min). Inset top: control without the CTR, no significant stabilization is observed without the CTR. Inset bottom: kinetics of DsrA_core_ stabilization by CTR. ∆CD refers to CD at a specific time subtracted from CD at t = 0.

**Figure 6:**
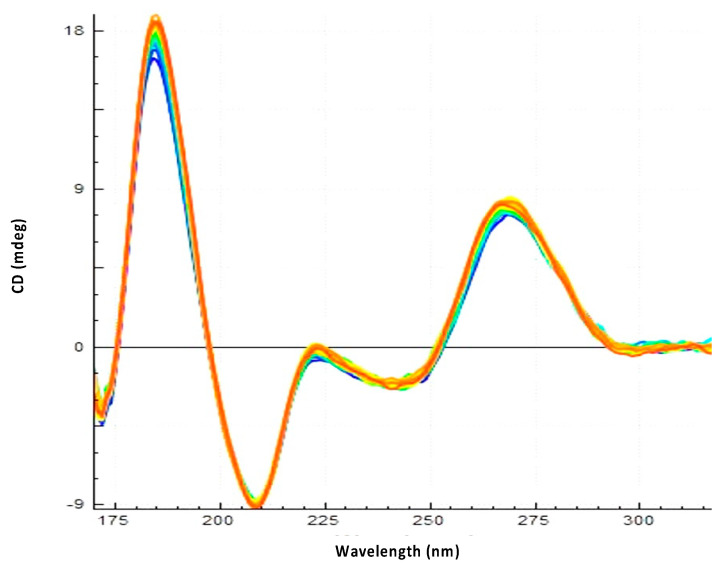
Effect of Hfq-CTR on DsrA_core_^mut^ spectral evolution. In this case no significant stabilization is observed. Note the amplitude of 18 mdeg measured at 180 nm. As DsrA_core_ (Figure 5) and DsrA_core_^mut^ were used at the same concentration and pathlength, this confirms that DsrA_core_^mut^ has a stronger (3-fold) structuration than DsrA_core_. This was expected due to the presence of the structured tetraloop.

One possibility is that annealing efficiency depends on the DsrA stem-loop which must be melted to associate with *rpoS*_reg_ (Figure 1). We thus tested the effect of Hfq-CTR on DsrA_core_ stability. Unexpectedly, we observed that Hfq-CTR does not melt DsrA_core_ SL1, but in contrast it stabilized it significantly (Figure 5). This can be observed by the increase of peaks at 180 and 270 nm (Figure 6) and by T_m_ measurements (Table 1). This is in contrast with DsrA_core_^mut^ where CTR does not have any effect (Figure 6). As the pentaloop of DsrA-SL1 is similar to single strand RNA (e.g., like AU_6_A in the linker between SL1 and SL2), we suspect this loop is important to form the complex. This is confirmed using EMSA where we show that Hfq-CTR binds DsrA_core_^mut^ but less than DsrA_core_ (Supplementary Materials Figure S4). The SL1 pentaloop is likely an important determinant for *rpoS* annealing to DsrA by Hfq-CTR, as it is the case of full Hfq [59].

### 3.3. Hfq CTR Does Not Affect rpoS Secondary Structure

**Figure 7:**
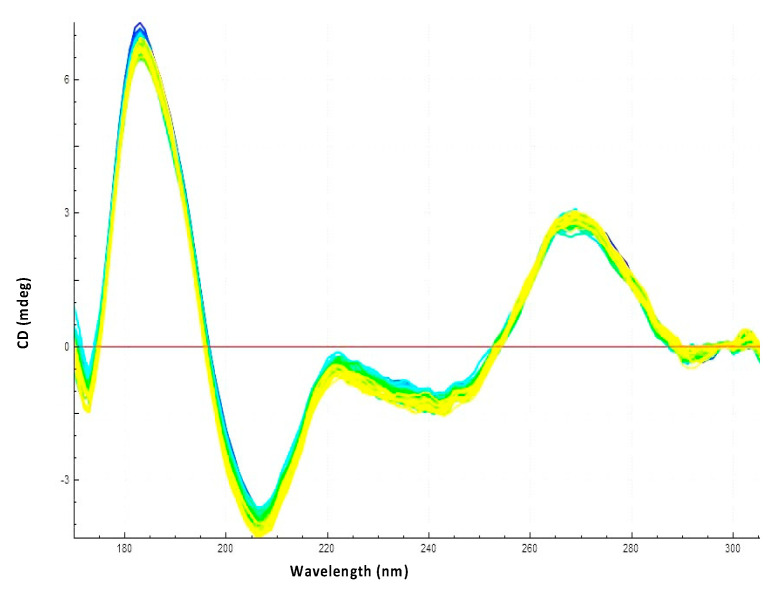
Observation of the effect of Hfq-CTR on *rpoS*_reg_:*rpoS*_rbs_ stability using SRCD (from blue t = 0 min to yellow t = 300 min). No significant stabilization is observed.

Another possibility to explain the effect of Hfq-CTR would be that it unwinds the *rpoS* stem-loop. This possibility was tested using a prehybrided *rpoS*_reg_:*rpoS*_rbs_. As shown in Figure 7, Hfq-CTR does not have any significant effect on the *rpoS*_reg_:*rpoS*_rbs_ complex and thus does not influence *rpoS* structure. This was also confirmed by T_m_ measurement; Hfq-CTR does not affect *rpoS*_reg_:*rpoS*_rbs_ stability (Table 1).

## 4. Discussion

Using SRCD analysis, we show herein that, unexpectedly, the amyloid region of Hfq-CTR is able to promote DrsA:*rpoS* annealing with an efficiency similar to that of the full-length protein at 15 °C. This work thus provides a new useful tool to analyze label-free RNA annealing by an amyloid protein, an analysis that is not trivial using classical EMSA (Supplementary Materials Figure S2). This spectroscopic analysis provides an alternative to fluorescence measurements without RNA labelling, that in addition gives information on base-pairing, base-stacking, and type of helix formed [72,73]. In turn, SRCD could also allow observation of the formation of the amyloid structures [45].

We show that the effect of Hfq-CTR is not due to the melting of the SL1 loop of DsrA (Figure 5) nor to the melting of the *rpoS* stem (Figure 6), which contrasts with the mechanism observed for the full Hfq including the Sm core [62]. Indeed, ribonuclease footprinting shows full Hfq binds the AUUUC DsrA pentaloop and that it melts DsrA-SL1 [62].

**Figure 8:**
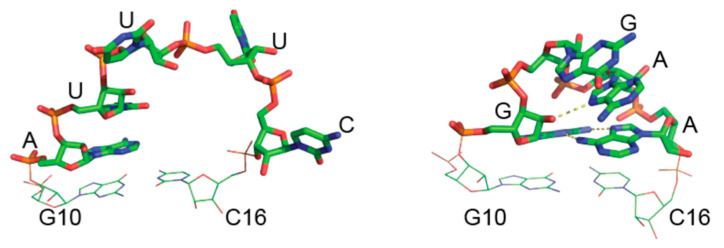
Structure of DsrA SL1 WT pentaloop (**left**) vs. mutated tetraloop (**right**). Note that G10 and C16 refers as WT DsrA SL1 numbering but should be 15 for the tetraloop.

As for the recognition of DsrA by Hfq-CTR, DsrA-SL1 contains a capping AUUUC pentaloop (Figure 8, left). All these five nucleotides are conserved in *Escherichia coli*, *Salmonella typhimurium,* and *Klebsiella pneumoniae*, and are all complementary to the *rpoS* mRNA. Previous mutational analysis of DsrA-SL1 showed that DsrA sRNA lost its activity to activate *rpoS* translation with most of the mutations in this pentaloop [54]. NMR studies show that A11 and U12 are stacked in a helical environment, while U13, U14, and C15 are highly flexible [59]. All five nucleotides of DsrA-SL1 participate in the formation of an A-form helix in the structure of the DsrA:*rpoS* complex. These studies suggest that the highly dynamic character of DsrA-SL1 plays an important role in base-pairing with *rpoS* mRNA and in regulating its translation [59]. To investigate the role of DsrA-SL1 in regulating *rpoS* translation, we thus designed a mutated form of DsrA_core_ called DsrA_core_^mut^ in which AUUUC pentaloop is replaced by GGAA tetraloop in DsrA-SL1. The GGAA tetraloop belongs to the GNRA tetraloop family (N = any nucleotide, R = A or G), which is extremely widespread, comprising one-third of the tetraloops in ribosomal RNA [74]. The GNRA tetraloop is particularly stable in comparison with other RNA loops [75]. The GGAA tetraloop forms an asymmetric loop structure, where only the first G is stacked on the 5′ side of the stem and the last three nucleotides stack on the 3′ side of the stem [76]. It also contains several hydrogen bonds. The first G and fourth A form a sheared base pair with two hydrogen bonds. Additional hydrogen bonding occurs between the 2′-OH of the first G and the base R7 of the third A [77]. The extensive base stacking and the intramolecular interactions provide GGAA tetraloops with a high thermodynamic stability. We also confirm this effect with our T_m_ measurement (Table 1).

Here we show that Hfq-CTR binds to WT DsrA SL1, and also to the mutated form of the SL1 with a tetraloop, but less solidly than WT (Supplementary Materials Figure S4, Figure 3 and Figure 6). We also show that the presence of the tetraloop in DsrA SL1 does not allow efficient annealing to *rpoS* (probably due to less base pairing with *rpoS*_reg_) and more important stabilization of DsrA (Figure 2 and Figure 6). Thus, we conclude that the effect of the CTR is directly linked to the efficiency of sRNA:mRNA annealing and not to complex stabilization nor SL1 melting. As Hfq-CTR binds the tetraloop mutant less than WT pentaloop, and cannot stabilize the mutant structure, it may indicate that CTR binds the AUUUC pentaloop and then promotes sRNA:mRNA annealing.

Finally, EMSA shows that Hfq-CTR can bind free DsrA_core_, free *rpoS*_reg_, and DsrA_core_:*rpoS*_reg_ and suggests that Hfq-CTR prefers to bind to the DsrA_core_:*rpoS*_reg_ complex (Supplementary Materials Figure S2). However, it has been shown that Hfq cannot bind the DsrA:r*poS* complex very well after annealing [68]. This result should be investigated further as it could indicate that the NTR/Sm domain of Hfq may transfer the DsrA:*rpoS* complex to CTR after annealing [40]. Unfortunately, it was not possible to analyze Hfq-NTR activity using SRCD due to the poor solubility of this form of the protein.

## 5. Conclusions

The main and unexpected result of this study is that an amyloid-like region promotes RNA annealing. This result opens a perspective for a new physiological role of amyloids, including those associated with neurodegenerative diseases. In addition, we show here that a biophysical method, SRCD, can be used to follow RNA annealing by an amyloid protein, which is not trivial using EMSA. The role of amyloids on deoxyribonucleic (DNA) and ribonucleic (RNA) acid conformational changes has been observed previously [78]. In particular, amyloids may bind non-coding RNA [79]. They also may sequester RNA [80]. As stress-induced RNA activates amyloidogenesis in vivo [81], the effect of stress induced sRNA such as DsrA on amyloidogenesis may also occur, such as with DNA [45], and should be investigated further.

## Figures and Tables

**Table 1:** Melting point (T_m_) of RNA measured at 180 nm. DsrA_core_ + *rpoS_reg_* means not prehybrided, DsrA_core_:*rpoS*reg means prehybrided by heat and cooling (see methods).

Sample	T_m_ at ~180 nm (°C)
DsrA_core_ + *rpoS*_reg_	33.6 ± 2.2
DsrA_core_ + *rpoS*_reg_ + CTR	36.6 ± 0.8
DsrA_core_ + *rpoS*_reg_ + Hfq	37.0 ± 0.9
DsrA_core_:*rpoS*reg	39.9 ± 0.4
DsrA_core_:*rpoS*_reg_ + CTR	44.2 ± 0.7
DsrA_core_	42.1 ± 0.3
DsrA_core_ + CTR	45.5 ± 0.8
DsrA_core_^mut^	49.1 ± 1.2
DsrA_core_^mut^ + CTR	50.3 ± 0.9
*rpoS*_rbs_ + *rpoS*_reg_	29.5 ± 2.2
*rpoS*_rbs_ + *rpoS*_reg_ + CTR	31.6 ± 1.7

## Data Availability

The SRCD data that support the findings of this study are available on request from the corresponding authors.

## Supplementary Materials

The following are available online at https://www.mdpi.com/article/10.3390/biology10090900/s1, Figure S1: Observation of the shift of the SRCD peak maximum (λ_max_) from ~180 to 190 nm for DsrA_core_ and DsrA_core_^mut^, Figure S2: EMSA analysis to confirm DsrA_core_ *rpoS*_reg_ annealing reaction by Hfq-CTR, Figure S3: Observation of DsrA_core_ and *rpoS_reg_* annealing by Hfq full-length using SRCD, Figure S4: Analysis of the complex between DsrA_core_^mut^ and CTR.

## Abbreviations

CTR/NTR	C/N-terminal region
IDP	Intrinsically Disordered Proteins
rbs	ribosome binding site
sRNA	small noncoding RNAs
SL	stem-loop
SRCD	Synchrotron Radiation Circular Dichroism.
