# Supplementary material for: The Amyloid Region of Hfq Riboregulator Promotes DsrA:rpoS RNAs Annealing
Source: Biology (Basel). 2021 Sep 12;10(9):900. doi: 10.3390/biology10090900 (PMC8468756; doi:10.3390/biology10090900)
Supplement: Supplementary file 1 [file biology-10-00900-s001.zip › biology-1355871-supplementary.pdf]

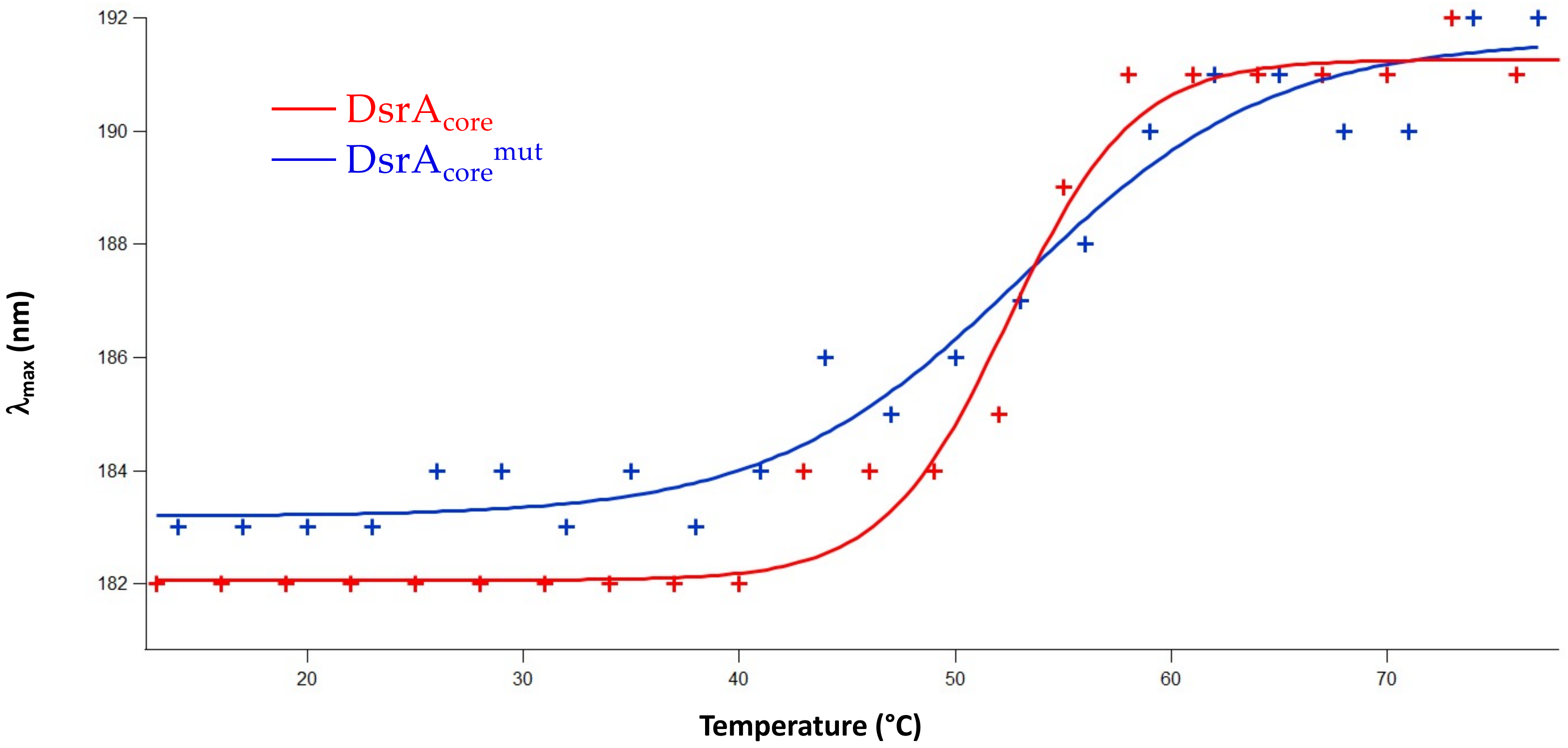

Sup. Fig. S1. Observation of the shift of the SRCD peak maximum (λ_{max}) from ~ 180 to 190 nm for $DsrA_{core}$ and $DsrA_{core}^{mut}$. Red: $DsrA_{core}$; blue $DsrA_{core}^{mut}$. In this case the T_m can be approximated at ~ 52 °C for $DsrA_{core}$ and ~ 53 °C for $DsrA_{core}^{mut}$, but can not be measured precisely to allow a comparison of RNAs stabilities.

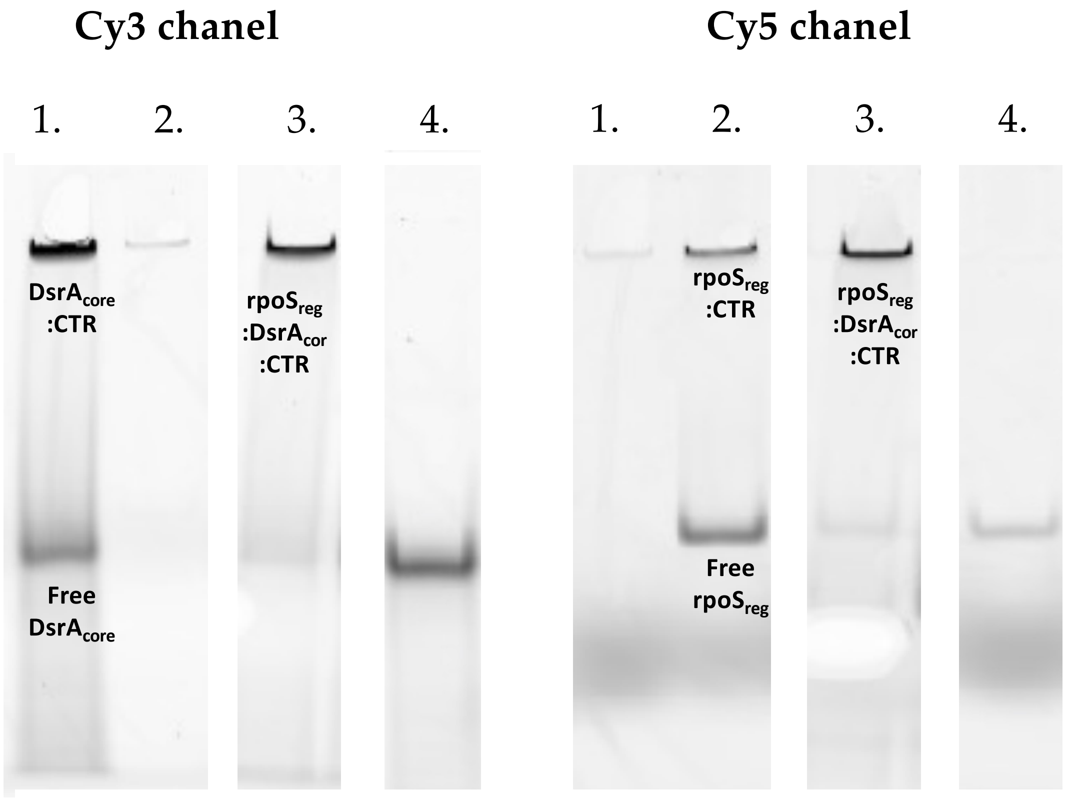

Sup. Fig. S2. EMSA analysis to confirm DsrA$_{core}$ *rpoS*$_{reg}$ annealing reaction by Hfq-CTR. Left panel Cy3 chanel; Right panel Cy5 chanel. lane 1: Cy3-DsrA$_{core}$ + CTR; Lane 2: Cy5-*rpoS*$_{reg}$ + CTR; Lane 3: Cy5-rpoS$_{reg}$ + Cy3-DsrA$_{core}$ + CTR after an incubation of 1h at 15°C. We observe the complex formed between CTR and RNAs on the top of the gel. Lane 4: Cy5-rpoS$_{reg}$ + Cy3-DsrA$_{core}$ without CTR.

EMSA tends to confirm that DsrA$_{core}$ and *rpoS*$_{reg}$ anneal in the presence of CTR. Nevertheless, due to the high molecular weight of the complex for the amyloid form of the CTR it migrates on the top of the gel and it is difficult to discriminate the complex CTR:Cy3-DsrA$_{core}$:Cy5-*rpoS*$_{reg}$ from CTR:Cy3-DsrA$_{core}$ and CTR:Cy5-*rpoS*$_{reg}$ (duplexes). This justifies the use of SRCD to confirm RNA annealing.

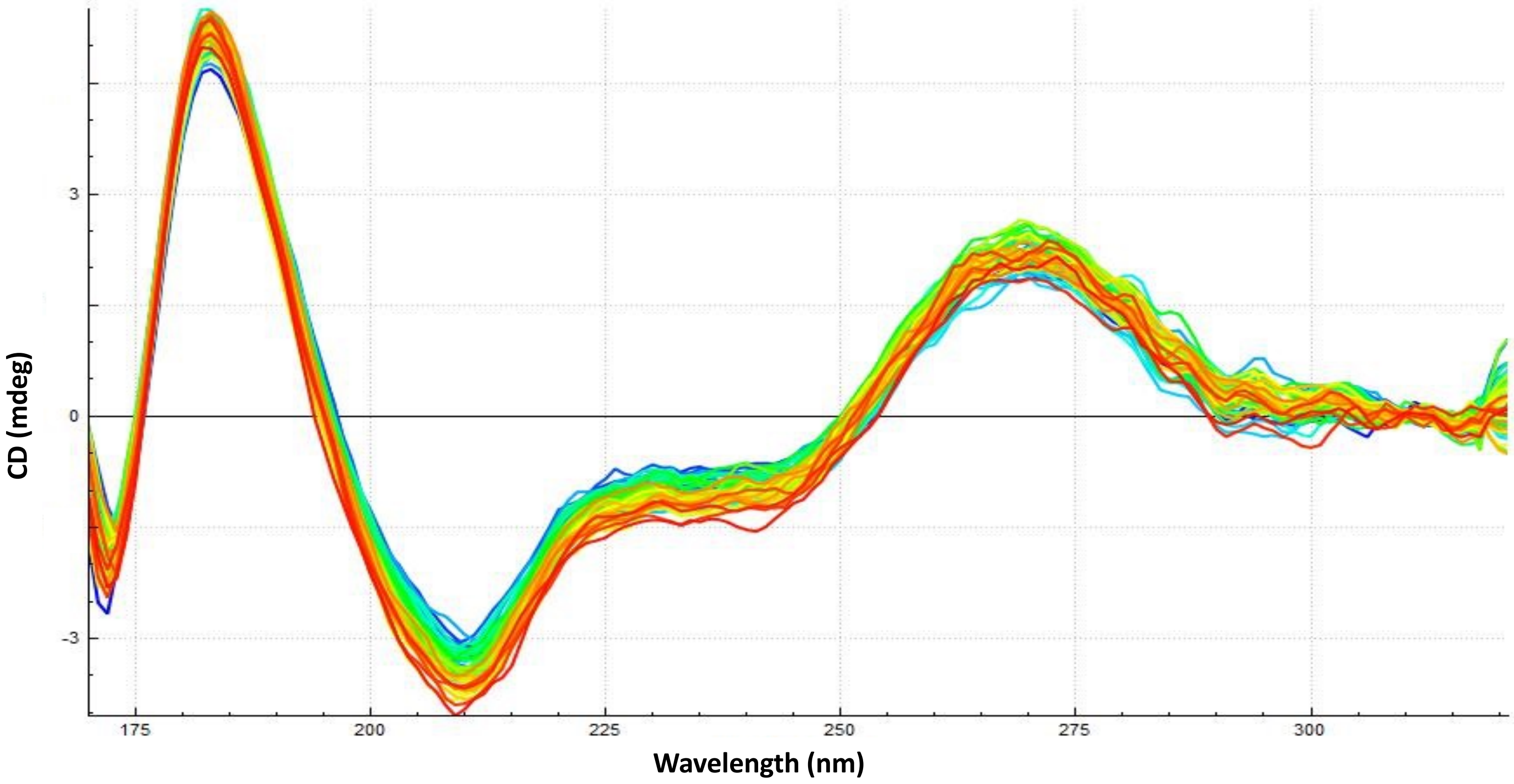

Sup. Fig. S3. Observation of $DsrA_{core}$ and $rpoS_{reg}$ annealing by Hfq full length using SRCD (from blue t=0 min to red t=325 min).

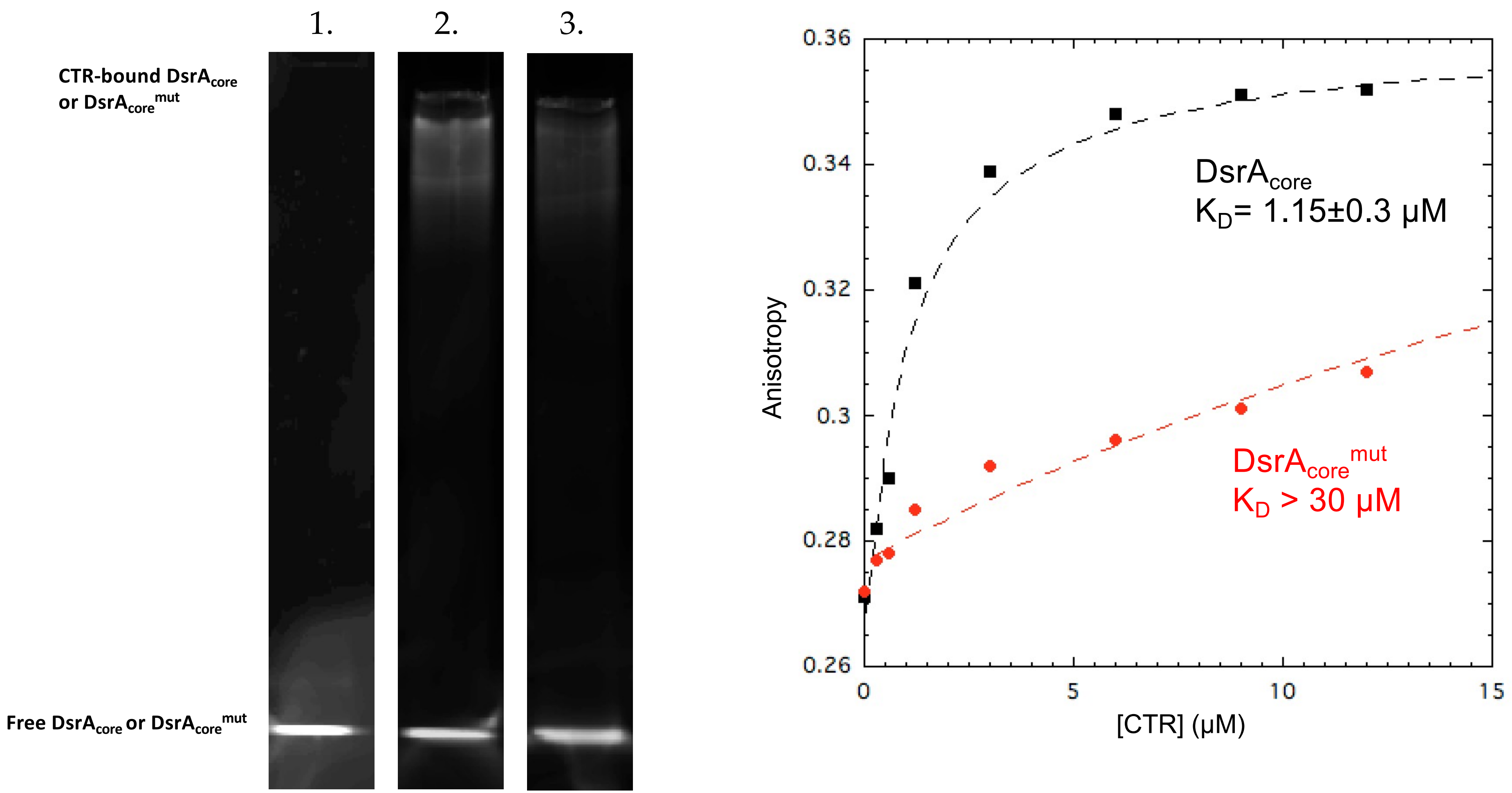

Sup. Fig. S4. (A) EMSA analysis of the complex between DsrA$_{core}^{mut}$ and CTR. Lane 1: DsrA$_{core}^{mut}$ without CTR, Lane 2 DsrA$_{core}$ with CTR, Lane 3: DsrA$_{core}^{mut}$ with CTR. As seen, CTR binds DsrA$_{core}^{mut}$ but to a lesser extent than DsrA$_{core}$. DsrA$_{core}$ and DsrA$_{core}^{mut}$ were stained using Gelred (Biotum). ***(B)*** Affinity of CTR for DsrA$_{core}$ and DsrA$_{core}^{mut}$ was then estimated using fluorescence anisotropy measurements (see protocol in Arluison *et al* Nucleic Acids Res (2007) 35:999-1006. K_D was around 1.15μM for DsA$_{core}$ while no saturation can be reached for DsrA$_{core}^{mut}$. In comparison, for full-length Hfq, K_D for DsrA$_{core}$ was previously estimated around 0.2 μM (see Arluison *et al* Nucleic Acids Res (2007) 35:999-1006)
